# The Genetics of Inherited Cholestatic Disorders in Neonates and Infants: Evolving Challenges

**DOI:** 10.3390/genes12111837

**Published:** 2021-11-21

**Authors:** Rebecca Jeyaraj, Kirsten McKay Bounford, Nicola Ruth, Carla Lloyd, Fiona MacDonald, Christian J. Hendriksz, Ulrich Baumann, Paul Gissen, Deirdre Kelly

**Affiliations:** 1 National Institute for Health Research Great Ormond Street Hospital Biomedical Research Centre, University College London, London WC1N 1EH, UK; rebecca.jeyaraj@kcl.ac.uk; 2 West of Scotland Centre for Genomic Medicine, Queen Elizabeth University Hospital, Glasgow G51 4TF, UK; kirstenmck@yahoo.com; 3 Institute of Immunology and Immunotherapy, University of Birmingham, Birmingham B15 2TT, UK; nicola.ruth1@nhs.net (N.R.); Baumann.U@mh-hannover.de (U.B.); deirdre@kellyda.co.uk (D.K.); 4 Liver Unit, Birmingham Women’s and Children’s Hospital, Birmingham B4 6NH, UK; carla.lloyd1@nhs.net; 5 West Midlands Regional Genetics Service, Birmingham Women’s and Children’s Hospital, Birmingham B15 2TG, UK; f.macdonald50@btinternet.com; 6 Steve Biko Academic Unit, Level D3 New Pretoria Academic Hospital, Malherbe Street, Pretoria 0002, South Africa; chris@fymcamedical.co.uk; 7 Paediatric Gastroenterology and Hepatology, Hannover Medical School, 30625 Hannover, Germany; 8 National Institute for Health Research Great Ormond Street Hospital Biomedical Research Centre, University College London, London WC1N 1EH, UK

**Keywords:** neonatal cholestasis, infantile cholestasis, next-generation sequencing, heterozygous pathogenic variants

## Abstract

Many inherited conditions cause cholestasis in the neonate or infant. Next-generation sequencing methods can facilitate a prompt diagnosis in some of these cases; application of these methods in patients with liver diseases of unknown cause has also uncovered novel gene-disease associations and improved our understanding of physiological bile secretion and flow. By helping to define the molecular basis of certain cholestatic disorders, these methods have also identified new targets for therapy as well patient subgroups more likely to benefit from specific therapies. At the same time, sequencing methods have presented new diagnostic challenges, such as the interpretation of single heterozygous genetic variants. This article discusses those challenges in the context of neonatal and infantile cholestasis, focusing on difficulties in predicting variant pathogenicity, the possibility of other causal variants not identified by the genetic screen used, and phenotypic variability among patients with variants in the same genes. A prospective, observational study performed between 2010–2013, which sequenced six important genes (*ATP8B1*, *ABCB11*, *ABCB4*, *NPC1*, *NPC2* and *SLC25A13*) in an international cohort of 222 patients with infantile liver disease, is given as an example of potential benefits and challenges that clinicians could face having received a complex genetic result. Further studies including large cohorts of patients with paediatric liver disease are needed to clarify the spectrum of phenotypes associated with, as well as appropriate clinical response to, single heterozygous variants in cholestasis-associated genes.

## 1. Introduction

Cholestasis refers to a reduction in bile flow as a result of impaired hepatocyte secretion or obstructed bile flow through the intrahepatic or extrahepatic bile ducts. In neonates and infants, cholestasis can occur due to a wide range of conditions which may have similar or overlapping presentations. This can make diagnosis based on clinical, biochemical, radiological and histological features challenging. In recent years, the decreased cost and increased availability of genetic technologies has led to the use of next-generation sequencing (NGS) methods to obtain a molecular diagnosis in neonates and infants with cholestasis of an otherwise indeterminate cause. These technologies have also facilitated the discovery of novel cholestasis-associated variants, such as variants in genes involved in the organisation of intercellular junctions [1] and intracellular trafficking [2]. This in turn has improved our understanding of bile acid physiology at the molecular level and informed modern therapeutic approaches to cholestasis.

Here, we aim to (1) briefly describe the sequence of events required for normal bile secretion and flow, (2) summarise the genetic causes of cholestasis in neonates and infants, (3) review the evolving role of sequencing technologies in the work-up of neonatal and infantile cholestasis, and (4) discuss challenges in the interpretation of single heterozygous pathogenic variants using data from an international multicentre project that involved sequencing of six cholestasis-associated genes in 222 patients with infantile liver disease.

## 2. Normal Bile Secretion and Flow

Bile is an aqueous mixture of bile salts, bilirubin, phospholipids, cholesterol, amino acids, steroids, enzymes, porphyrins, vitamins and heavy metals [3]. It may also contain exogenous drugs, xenobiotics and environmental toxins [3]. Bile is first secreted by the hepatocyte into the canalicular lumen, where biliary flow is aided by transcellular and paracellular fluid movement as well as peristaltic actin contractions [4]. This bile is then modified by secretory and absorptive processes in the bile duct epithelium as it flows distally through the bile ducts. More specifically, transport proteins within the luminal membrane of cholangiocytes secrete bicarbonate and chloride while reabsorbing fluid and solutes such as glucose, glutamate and urate from the original secretion of hepatocytes [5]. This modified bile can then be stored within the gallbladder or secreted into the duodenal lumen.

Impairment of bile secretion and flow compromises the emulsification, digestion and absorption of dietary lipids as well as the excretion of cholesterol and other substances [6]. In addition, bile can accumulate within the liver and systemic circulation where it may exert toxic effects through a detergent action on cellular lipid components and the generation of reactive oxygen species [7]. As bile acids also act as signalling molecules that modulate gene expression, epithelial cell proliferation and glucose and lipid metabolism [6], these processes may be affected by impaired bile flow.

## 3. Genetic Causes of Cholestasis in Neonates and Infants

Where any of the molecular events required for normal bile secretion and flow are disrupted, cholestasis can develop. The single most common cause of cholestasis in neonates and infants is biliary atresia (BA), a deficiency of the extrahepatic bile ducts with an unclear aetiology [8]. Following this, it is estimated that 25–50% of cases of cholestasis occur due to identifiable genetic mutations [9,10,11,12]. These mutations involve a wide variety of genes which have either a direct or indirect effect on the synthesis, transport and flow of bile. Among the more commonly implicated genetic and metabolic diseases are α1-antitrypsin (A1AT) deficiency and Alagille syndrome. Neonatal intrahepatic cholestasis due to citrin deficiency (NICCD) is thought to be more common in East Asian populations [13,14,15], while Niemann–Pick disease type C1 (NPC-1) may be more common in certain isolated populations such as the Hispanic population from New Mexico [8,16]. Examples of genetic disorders caused by such mutations are summarised in Table 1.

Other non-genetic causes of cholestasis include hypopituitarism, biliary sludge, preterm birth, parenteral feeding and infection. Some neonates with a history of an injurious event who develop a cholestasis that resolves clinically and biochemically during follow-up are described as having ‘transient neonatal cholestasis’ (TNC). With the more frequent use of sequencing methods to obtain a molecular diagnosis in this age group, there has been a decrease in the proportion of patients classified as having TNC [35].

## 4. The Role of Next-Generation Sequencing in the Work-Up of Cholestasis

**Figure 1:**
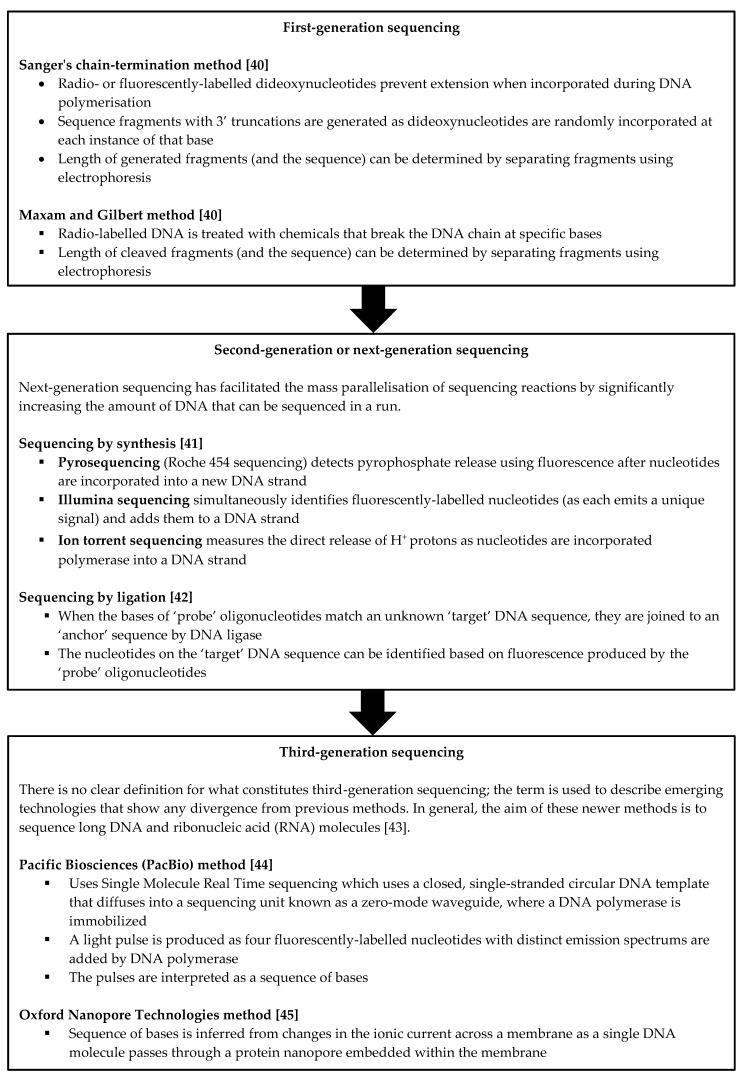
Overview of past, current and emerging sequencing methods. [40,41,42,43,44,45].

NGS has an increasingly important role in the work-up of neonatal and infantile cholestasis. Its rapid and high-throughput nature allows for the identification of variants from targeted gene panels, the whole exome or the whole genome [36]. This has made the discovery of novel cholestasis-associated genes and variants possible. It has also allowed for a molecular diagnosis to be obtained in patients with liver disease of an otherwise uncertain cause; the ability to obtain a diagnosis in these cases can ensure that appropriate therapies are instituted while therapies which do not offer benefit are avoided. For instance, liver transplantation is not generally associated with favourable outcomes in conditions such as mitochondrial deoxyribonucleic acid (DNA) depletion syndrome [37,38] or NPC [39]. A brief description of past, current and emerging sequencing methods is provided in Figure 1.

Given the cost-effectiveness of NGS techniques, genetic testing may be used increasingly early on in the diagnostic process. At the same time, the use of genetic testing too early or without selection could give rise to unnecessary or uninterpretable information that complicates rather than clarifies the diagnosis. Feldman and Sokol in fact describe a proposed algorithm for the work-up of neonatal cholestasis that seems to balance these considerations, with genetic testing via a targeted gene panel or whole-exome sequencing potentially occurring once BA, A1AT deficiency and red flags warranting specific evaluation are excluded [8].

## 5. New Diagnostic Challenges in the Era of Sequencing

NGS has allowed for the characterisation of several autosomal recessive conditions that cause cholestasis. In these conditions, patients must usually have two affected alleles in order to develop the disease. Recently, it has been proposed that even heterozygous changes in certain genes may predispose to cholestatic disease, particularly where a second challenge is present, such as drugs, hormones or inflammatory mediators. For instance, TNC has been linked to heterozygous pathogenic variants in the *ATP8B1*, *ABCB11* and *ABCB4* genes which are involved in the transport of bile [46,47].

In general, heterozygous pathogenic variants can cause disease through three mechanisms [48]. First, single gain-of-function variants may result in the production of an altered gene product with a new molecular function or may alter the pattern of gene expression. Second, a heterozygous pathogenic variant may exert a dominant negative effect, where the altered gene product interferes with the function of the wild-type gene product. Third, a heterozygous loss-of-function variant can lead to haploinsufficiency, where the dosage of normal gene product produced by the single remaining wild-type allele is not sufficient to sustain a normal phenotype.

### 5.1. Frequency of Heterozygous Pathogenic Variants in Children with Undiagnosed Liver Disease

While there are known biological mechanisms through which heterozygous pathogenic variants can cause disease, the attribution of clinical significance to these variants is not straightforward.

To illustrate the challenge of determining the clinical significance of heterozygous variants, we use our own data from a previously unpublished multicentre study. From 15 January 2010 to 16 January 2013, sequencing data from 222 children under 2 years of age were obtained across 12 different countries (Bulgaria, Canada, Denmark, Germany, Greece, Hungary, India, the Netherlands, Oman, Poland, Turkey and the UK). A microarray resequencing (MS) method was used to sequence DNA from 44 patients as published previously [49], while NGS with the GS Junior platform (Roche, Branford, CT, USA) was used for the remaining 178 patients. The genes of interest which were sequenced at the time of the study were: *ATP8B1*, *ABCB11*, *ABCB4*, *NPC1*, *NPC2* and *SLC25A13*. Sequence variants identified by the MS and NGS tests were confirmed using Sanger sequencing to minimise false positive calls. Variant interpretation was performed using Alamut v2.1, with variants being classified as ‘pathogenic’, ‘possibly pathogenic’, ‘variant of uncertain significance’, ‘possibly benign’ or ‘benign’. Follow-up information about the status of liver disease was obtained between 1 and 2 years after initial recruitment to the study. Informed consent was obtained from all participating families, and the study protocol was approved by all relevant institutional ethics committees. Further details can be found in reference [50].

The children included in the study presented with: cholestasis as determined by serum conjugated bilirubin levels > 20 μmol/L or > 20% of total bilirubin (*n* = 212), acute liver failure as determined by prothrombin time more than twice the upper limit of normal for age (*n* = 39), and hepatomegaly (*n* = 137) and/or splenomegaly (*n* = 99) as observed on clinical or ultrasound examination. Patients were not included if a family member had been diagnosed with a genetic condition known to cause neonatal cholestasis. Across the whole cohort, 19 patients (8.5%) were diagnosed with NPC-1, progressive familial intrahepatic cholestasis (PFIC) types 1–3 or NICCD by identification of two changes in the same gene that were determined to be pathogenic or possibly pathogenic (Table 2). Single heterozygous variants predicted to be pathogenic or possibly pathogenic (hereafter referred to as mutations) were identified in 20 patients (i.e., 9% of included patients) and are summarised below as well as in Table 3.

#### 5.1.1. *ATP8B1*, *ABCB11* and *ABCB4* Mutations

*ATP8B1* encodes a P-type ATPase flippase that translocates phospholipids from the outer to the inner leaflet of the hepatocanalicular membrane bilayer. Biallelic mutations in this gene can lead to PFIC type 1 [54]. In our cohort, five patients were identified with single heterozygous mutations in *ATP8B1* (Table 3). All five presented with cholestasis; one patient also had hepatomegaly, and another had hepatosplenomegaly and acute liver failure. Progressive liver disease led to liver transplantation in three patients, although BA was cited as an alternative diagnosis in two of these cases. The symptoms of the remaining two cases were reported to have resolved.

*ABCB11* encodes the bile salt export pump (BSEP) and has been implicated in PFIC type 2. In our cohort, four patients were found to have single heterozygous mutations in *ABCB11*; one of these also had a possibly pathogenic change in *ABCB4* (Table 3). Symptoms resolved without intervention in two of these cases, one patient improved after a partial external biliary diversion procedure, and no information was available for the fourth patient.

*ABCB4* encodes the multidrug resistance protein 3 (MDR3), a floppase that translocates phospholipids from the inner to the outer leaflet of the hepatocanalicular membrane bilayer. Pathogenic changes in this gene can lead to PFIC type 3. In our cohort, single heterozygous *ABCB4* mutations were identified in three patients, excluding the aforementioned patient with mutations in both *ABCB11* and *ABCB4* (Table 3). All three patients presented with cholestasis: two also had splenomegaly, one had hepatomegaly, and one had acute liver failure. One had BA requiring a liver transplant, and one died at 15 months of age due to multi-organ failure.

#### 5.1.2. *NPC1* and *NPC2* Mutations

*NPC1* and *NPC2* encode an intracellular cholesterol transporter that facilitates the egress of cholesterol from lysosomes. Biallelic mutations in these genes cause Nieman-Pick disease type C1 and C2, respectively.

Seven patients were identified with a single heterozygous mutation in the *NPC1* gene (Table 3). All seven patients had cholestasis: five had hepatomegaly, two had splenomegaly, and one had acute liver failure at presentation. In four patients, visceral symptoms resolved with no intervention. One patient with progressive liver disease was given an alternative diagnosis of BA and required a liver transplant. The remaining two patients, however, had progressive liver disease with no alternative diagnosis.

Single mutations in the *NPC1* gene have not previously been associated with infantile cholestatic disease. However, a previous study of the *NPC1* and *NPC2* genes in adults with neurological features identified several patients with single mutations [63]. Bauer and colleagues raised the possibility of a late-onset, reduced-penetrance form of Nieman-Pick disease type C. In addition, heterozygous mutations in *NPC1* have been associated with obesity in adulthood [64,65]. It would be useful to investigate a cohort of *NPC1* mutation carriers to help determine whether there is an increased risk of infantile liver disease or adult-onset neurological or metabolic problems in these patients.

No patients were found to have single heterozygous mutations in the *NPC2* gene.

#### 5.1.3. *SLC25A13* Mutations

*SLC25A13* encodes citrin, a calcium-dependent mitochondrial solute transporter which plays a role in the aspartate-malate reduced nicotinamide adenine dinucleotide shuttle and the urea cycle. Biallelic mutations in this gene cause NICCD. One patient with a single heterozygous mutation in *SLC25A13* was identified in our cohort (Table 3). This patient had cholestasis and hepatosplenomegaly, which resolved without intervention. NICCD would be predicted to resolve in infancy, so the significance of this finding is not yet clear.

### 5.2. Difficulties in Predicting Pathogenicity

Having described the clinical features in children with single heterozygous pathogenic variants in six pre-specified genes, it is now important to consider why assigning causality to these variants is not straightforward.

The first challenge lies in accurately predicting the pathogenicity of identified variants. At the time of our study, 13 out of 23 (57%) of the unique sequence variants detected among patients diagnosed with autosomal recessive PFIC and NPC were considered novel. This suggests that many of these rare variants may be family-specific and therefore external information about them may not be available. Ideally, the discovery of potential pathogenic variants would be followed up by further studies to better characterise their cellular effects. In vitro studies are particularly useful here as these studies can often provide a more complete characterisation of genetic variants compared to bioinformatic prediction alone. For instance, case reports of individual patients which combine genetic findings with findings from histological examination or other in vitro experiments can provide valuable insights. Baghdasaryan and colleagues relate a single heterozygous missense variant in *ABCB11* to a histologically proven reduction in BSEP expression in a young patient with TNC, providing a useful genotype-phenotype correlation [66]. Zhang and colleagues describe transcriptional and functional studies in a patient with NICCD and compound heterozygous variants in *SLC25A13*; reverse transcriptase-polymerase chain reaction studies suggested that one variant was a splice-site variant resulting in intron retention, while a yeast model showed that the other novel missense variant led to complete loss of the aspartate-glutamate carrier function of citrin [67]. Broader studies have also been undertaken to evaluate multiple genetic variants using in vitro assays—for example, Park et al. investigated the effect of eight *ABCB4* pathogenic variants on MDR3 transport activity and plasma membrane expression in both the presence and absence of a pharmacological chaperone [68]. They found, unexpectedly, that only three of the variants led to reduced transport activity, and were able to distinguish reduced transport activity due to reduced plasma membrane expression from reduced activity with maintained expression. They additionally found that a pharmacological chaperone could restore expression and function for certain trafficking-defective *ABCB4* variants. Overall, these studies focusing on fundamental research offer a unique lens through which gene-disease associations and therapeutic possibilities can be interrogated.

However, large-scale genetic studies are likely to yield too many variants for each variant to be studied in-depth. Bioinformatic tools therefore remain a useful method by which pathogenicity might be predicted. That said, it is important to recognise that these prediction tools could still incorrectly classify a variant as pathogenic when in fact it does not contribute to the disease. This is because variant interpretation requires information beyond the predicted effect on protein function—for instance, variants must be interpreted alongside information on gene-disease validity, pattern of inheritance, allele frequency, the most clinically relevant transcript, and underlying pathophysiological mechanisms [69]. It is therefore possible for variants classified as pathogenic using bioinformatic tools to be benign, and to potentially be reclassified as such as more information becomes available over time.

Interestingly, a significant proportion (19%) of our patients without pathogenic variants was found to have variants of uncertain significance or possibly benign variants. Again, it is possible for these variants to be reclassified as more information regarding their association (or lack thereof) with disease becomes available through future studies and long-term follow-up of outcomes. Trio sequencing—that is, sequencing of the patient in addition to their biological parents—can also be useful when variants of uncertain significance are encountered as this allows determination of which variants are *de novo* and which are inherited from the parents. This in turn facilitates variant interpretation where phenotype information from the parents is available.

Within the scope of variant pathogenicity, it is also worth considering the effect of heterozygous variants on liver injury in those who are susceptible to, or already have, more common types of liver disease. The milder PiMZ or PiSZ genotypes for A1AT deficiency, for instance, may exacerbate liver injury in patients who have other risk factors or comorbidities, such as those with alcohol-related liver disease [70], metabolic dysfunction-associated liver disease [70] or cystic fibrosis-related liver disease [71]. Similarly, the clinical significance of single heterozygous variants in the six genes we discuss above may in fact depend on whether a “second hit” is present, and this should be taken into account when predictions regarding variant pathogenicity are made.

A further challenge to understanding variant pathogenicity lies in the complex post-translational regulation that is necessary for the normal trafficking, subcellular localisation and function of proteins. For example, it is recognised that post-translational regulation in the form of protein-protein interaction is needed for efficient targeting of BSEP to the hepatocanalicular membrane [72]. Consideration of the wider BSEP interactome is therefore necessary when making conclusions about variant pathogenicity, since pathogenic variants in the *ABCB11* gene may affect interaction of the encoded BSEP protein with any of its molecular partners. Other work has also attempted to clarify the interaction partners of the MDR3 protein encoded by *ABCB4*. As an example, RAB10 belongs to the Ras-related in brain (RAB) family of proteins which serve as master regulators for vesicular transport, and has recently been identified as an interaction partner of MDR3 [73]. Ben Saad et al. showed that experimental silencing of RAB10 led to reduced plasma membrane localisation of MDR3 and reduced phosphatidylcholine secretion into bile. Again, this demonstrates that accurate predictions regarding a particular variant’s pathogenicity will depend in part on efforts to unpick the complex network of molecular interaction partners for the protein of interest.

### 5.3. Mutations Elsewhere That Have Not Been Identified

Although the combined incidence of PFIC, NPC, and NICCD cases in our cohort was difficult to assess because of their rarity, possible under-diagnosis, regional variation, and because the children had already been referred to a specialist centre, a conservative estimate would be that one case arises in every 50,000–100,000 live births. Based on the Hardy-Weinberg equilibrium, the expected incidence of mutation carriers in the general population is predicted to be around 1 in 110–160 people. The findings in this cohort (20/222, or approximately 1:11) therefore suggest an apparent increase in the prevalence of mutation carriers, which might be due to the sample size, misclassification of variants as pathogenic, case selection or because patients had other mutations that were not identified by the genetic screen.

The interpretation of heterozygous variants is thus complicated by the possibility of other mutations that are not picked up by the genetic screen used. These mutations may occur in genes which are not included in a targeted gene panel or have not yet been associated with the disease in question. For instance, recent case reports and case series have described patients with newly recognised types of infantile intrahepatic cholestasis caused by homozygous mutations in genes such as the *LSR* gene (involved in tricellular tight junction formation) [74] or the *KIF12* gene (involved in hepatocyte polarity) [75]; these genes would not be included in most diagnostic cholestatic gene panels in clinical use. Sequencing methods also do not detect all types of genetic variation—for instance, they may not detect large-scale deletions, duplications, repeat expansions or chromosomal rearrangements. Additionally, not all testing strategies allow for the detection of mutations in promoter or intronic regions which can also have important effects on cellular function.

With reference to the previously mentioned patient in Section 5.1.1 who had both a single heterozygous pathogenic change in *ABCB11* and a possibly pathogenic change in *ABCB4*, it is worth noting that the role of digenic heterozygosity has been discussed in the context of several other diseases [76,77,78,79]. In the context of genetic cholestasis, a recent case report described the finding of heterozygous digenic mutations in *ABCB4* and *ABCB11* in an infant with low phospholipid-associated cholelithiasis (LPAC) and TNC, where ursodeoxycholic acid led to resolution of symptoms [80]. However, without further large-scale studies, the broader importance of digenic heterozygosity in genetically determined cholestatic conditions is unclear.

### 5.4. Variable Clinical Phenotypes

The challenge of interpreting heterozygous mutations is further compounded by phenotypic variability among patients, as seen in the variable disease course in our patients with heterozygous changes. Common reasons for phenotypic variability include incomplete penetrance, where not all individuals with a particular genotype exhibit the disease phenotype, and variable expressivity, where individuals with a particular genotype exhibit different “degrees” of the disease phenotype. While the underlying basis for incomplete penetrance and variable expressivity are not certain, they are thought to arise due to the effect of other genetic factors (such as the mutation type or modifier genes), as well as epigenetic factors and hormonal and environmental influences.

With respect to *ATP8B1*, *ABCB11* and *ABCB4*, it seems increasingly likely that pathogenic changes in these genes may be implicated in a whole spectrum of disease, ranging from the severe progressive cholestatic disease seen in PFIC to intermittent forms such as benign recurrent intrahepatic cholestasis (BRIC), drug-induced cholestasis (DIC), intrahepatic cholestasis of pregnancy (ICP) and LPAC. For instance, PFIC and BRIC are both typically caused by biallelic mutations in *ATP8B1* or *ABCB11*; however, patients with BRIC do not exhibit the severe liver disease seen in patients with PFIC. This is thought to be related to the type of mutations present in each patient and their varying effects on protein expression and function [54,81]. Interestingly, Stättermayer and colleagues describe a brother and sister pair with the same homozygous variants in *ABCB4*, where the brother developed decompensated cirrhosis by the age of 38 while the sister (subsequently identified through family screening) was asymptomatic with normal liver biochemistry at the age of 34 [82]. This observation again suggests that in addition to the variant itself, other factors may have a role in determining the natural disease course in patients with *ABCB4* variants. Heterozygous mutations, particularly in *ABCB4*, have also been observed in some women with ICP, and it has been suggested that the increase in reproductive hormones in the later stages of pregnancy in combination with the heterozygous genotype may contribute to the predisposition to ICP in this group [83].

The importance of clarifying the relationship between genotype and phenotype becomes especially important when considering therapeutic intervention. Mutations in *ABCB11* which cause varying degrees of BSEP deficiency provide an illustrative example. Biallelic truncating mutations in this gene result in extremely low protein levels, and also more severe phenotypes compared to biallelic non-truncating mutations [84]. A retrospective cohort study found that less severe *ABCB11* mutations were associated with increased native liver survival, as well as lower serum bile acid levels and more frequent resolution of pruritis following surgical biliary drainage [85]. Recent data from the open-label phase 2 INDIGO trial further showed that patients with less severe (non-truncating) mutations who received the ileal bile acid transporter (IBAT) inhibitor maralixibat were more likely to experience a significant reduction in serum bile acid levels compared to patients with severe (truncating) mutations who received the same weight-adjusted dose [86]. These data underscore the importance of further studies aimed at establishing meaningful genotype-phenotype links in inherited cholestatic disorders, as this may not only predict natural disease course but also guide therapeutic strategies.

### 5.5. Practical Considerations

It is important to acknowledge that uncertainties in genetic diagnosis are often accompanied by a range of practical and ethical considerations [87]. For example, where the pattern of inheritance of a disease is unclear, screening and counselling of family members for the same or related conditions becomes difficult. As an example, where an infant with cholestasis is found to have a heterozygous pathogenic variant in a gene also known to be associated with ICP, appropriate medical advice for family members—including those who may be considering pregnancy—should be developed.

Moreover, as discussed above, our understanding of the importance of certain variants will change with time as more information becomes available. While variants initially considered to be pathogenic may be reclassified as benign, it is also possible that variants of uncertain significance subsequently become classified as pathogenic. This raises issues regarding follow-up and advice on risk factor avoidance. In some instances, clinicians and/or patients may consider re-contacting the testing laboratory periodically for updates [88]. Alternatively, as a matter of policy, a robust system for disseminating new clinically relevant information regarding pathogenic variants to patients may be considered.

Finally, where there is uncertainty in the interpretation of an individual’s genetic information, the potential for future genetic discrimination is important to consider [89]. Issues relating to disclosure of genetic test results to employers and insurers may arise and are further complicated by uncertainty regarding the clinical importance of the heterozygous genotype.

As sequencing methods become more commonly employed for both diagnostic and predictive purposes in paediatric hepatology centres across different countries, an awareness and discussion of the practical challenges posed by these technologies will be increasingly necessary to facilitate their integration into standard clinical practice.

## 6. Conclusions

In summary, the sequence of events required for normal bile secretion and flow can be disrupted by a wide range of pathogenic variants, resulting in cholestasis in the neonate or infant. The detection of such variants has been made increasingly convenient with the use of next-generation sequencing methods. The majority of diseases resulting from these variants seem to show what is traditionally considered to be an autosomal recessive inheritance pattern. However, there is growing evidence that single heterozygous pathogenic variants may also predispose to disease. The interpretation of these heterozygous variants is complicated by uncertainties in predicting pathogenicity, the possibility of unidentified causal variants elsewhere, as well as the observed phenotypic variability among patients. To address these challenges, correlation of clinical, biochemical and genetic findings in the individual patient remains essential. In addition, further studies to determine the cellular effects of different variants where possible, an inclusive registry to follow up patients with heterozygous changes over time, as well as retrospective analyses of neonatal phenotypes in BRIC, ICP, DIC and LPAC patients could help clarify the spectrum of cholestatic disease associated with single heterozygous pathogenic variants. Ultimately, clear genotype-phenotype correlations in these conditions could help predict natural disease course as well as the response to medical and surgical therapies.

## Figures and Tables

**Table 1:** Genetic disorders which can cause cholestasis in neonates and infants.

Mechanism	Examples (Not Exhaustive)	References
Biliary tract anomalies	Alagille syndrome	[17]
Defect in the synthesis of components of bile	**Bile acid synthesis defects** e.g., cerebrotendinous xanthomatosis (or sterol 27-hydroxylase deficiency) **Cholesterol synthesis defects** e.g., lathosterolosis	[18,19]
Defects in intracellular trafficking	Arthrogryposis-renal dysfunction-cholestasis syndrome *MYO5B*-associated intrahepatic cholestasis	[2,20]
Defects in the export of components of bile or in tight junction formation	Progressive familial intrahepatic cholestasis types 1—6 Rotor syndrome Dubin-Johnson syndrome	[21,22,23,24,25,26,27,28]
Metabolic disorders	**Disorders of lipid metabolism** e.g., Niemann-Pick disease type C, Wolman disease, Farber disease, Gaucher disease, cholesterol ester storage disease **Disorders of carbohydrate metabolism** e.g., galactosaemia, hereditary fructose intolerance, glycogen storage disease type IV **Disorders of amino acid metabolism** e.g., tyrosinaemia **Peroxisomal disorders** e.g., Zellweger syndrome, adrenoleukodystrophy **Urea cycle disorders** e.g., arginase deficiency, citrin deficiency **Mitochondrial disorders** e.g., mitochondrial DNA depletion syndrome, fatty acid oxidation defects, GRACILE (Growth Retardation, Aminoaciduria, Cholestasis, Iron overload, Lactic acidosis and Early death) syndrome	[29,30,31,32,33]
Miscellaneous disorders	α1-antitrypsin deficiency Cystic fibrosis Indian childhood cirrhosis Cholestasis of North American Indians Trisomy 13, 18, or 21 Turner syndrome	[10,29,30,31,32,34]

**Table 2:** Patients diagnosed with autosomal recessive conditions.

Patient	Mutation 1	Novel or Reference	Prediction Tools	Mutation 2	Novel or Reference	Prediction Tools	Diagnosis	Presenting Features	Features at Follow-Up
20	*NPC1* c.2000C>T p.(S667L)	[51]	AGVGD C65 SIFT Deleterious PP Probably damaging SSF No changes MES No changes NNS No changes GS Cryptic acceptor HSF No changes	*NPC1* c.3182T>C p.(I1061T)	[52] (rs80358259)	AGVGD C25 SIFT Deleterious PP Possibly damaging SSF No changes MES No changes NNS No changes GS No changes HSF No changes	NPC	C; H	Not available
40	*ATP8B1* c.1244A>G p.(Q415R)	Novel at time of study. Subsequently published in [53]	AGVGD C0 SIFT Deleterious PP Probably damaging SSF No changes MES No changes NNS No changes GS No changes HSF No changes	*ATP8B1* c.1244A>G p.(Q415R)	Novel at time of study. Subsequently published in [53]	AGVGD C0 SIFT Deleterious PP Probably damaging SSF No changes MES No changes NNS No changes GS No changes HSF No changes	PFIC type 1	C; H	Not available
86	*ATP8B1* c.1367C>T p.(T456M)	[54] (rs121909104)	AGVGD C0 SIFT Deleterious PP Probably damaging SSF No changes MES No changes NNS No changes GS No changes HSF No changes	*ATP8B1* c.2083G>A p.(E695K)	Novel	AGVGD C55 SIFT Deleterious PP Probably damaging SSF No changes MES No changes NNS No changes GS No changes HSF No changes	PFIC type 1	C; H	PEBD at 7 m C resolved
29	*ATP8B1* c.2788C>T p.(R930*)	[54] (rs140407614)	Nonsense mutation predicted to result in nonsense-mediated decay	*ATP8B1* c.2788C>T p.(R930*)	[54] (rs140407614)	Nonsense mutation predicted to result in nonsense-mediated decay	PFIC type 1	C	LT Current symptoms unknown
213	*ABCB11* c.731_732insA p.(I245Tfs*26)	Novel	Nonsense mutation predicted to result in nonsense-mediated decay	*ABCB11* c.779G>A p.(G260D)	Novel	AGVGD C0 SIFT Deleterious PP Probably damaging SSF No changes MES No changes NNS No changes GS No changes HSF No changes	PFIC type 2	C; H	PEBD at 14 m Pruritis resolved C persists
130	*ABCB11* c.1081C>T p.(Q361*)	Novel	Nonsense mutation predicted to result in nonsense-mediated decay	*ABCB11* c.1445A>G p.(D482G)	[23] (rs72549402)	AGVGD C65 SIFT Deleterious PP Probably damaging SSF No changes MES Cryptic donor NNS No changes GS No changes HSF Cryptic donor	PFIC type 2	C; S	PEBD at 14 m C persists
173	*ABCB11* c.1084-2A>G	Novel	SSF acceptor destroyed; cryptic acceptor MES acceptor destroyed; cryptic acceptor NNS acceptor destroyed; cryptic acceptor GS acceptor destroyed; cryptic acceptor HSF acceptor destroyed	*ABCB11* c.1084-2A>G	Novel	SSF acceptor destroyed; cryptic acceptor MES acceptor destroyed; cryptic acceptor NNS acceptor destroyed; cryptic acceptor GS acceptor destroyed; cryptic acceptor HSF acceptor destroyed	PFIC type 2	C; S; H; ALF	Not available
93	*ABCB11* c.1409G>A p.(R470Q)	[55]	AGVGD C35 SIFT Deleterious PP Probably damaging SSF No changes MES No changes NNS No changes GS No changes HSF No changes	*ABCB11* c.1409G>A p.(R470Q)	[55]	AGVGD C35 SIFT Deleterious PP Probably damaging SSF No changes MES No changes NNS No changes GS No changes HSF No changes	PFIC type 2	C; H	LT Asymptomatic post-transplant
180	*ABCB11* c.1409G>A p.(R470Q)	[55]	AGVGD C35 SIFT Deleterious PP Probably damaging SSF No changes MES No changes NNS No changes GS No changes HSF No changes	*ABCB11* c.1409G>A p.(R470Q)	[55]	AGVGD C35 SIFT Deleterious PP Probably damaging SSF No changes MES No changes NNS No changes GS No changes HSF No changes	PFIC type 2	C; S; H	Not available
74	*ABCB11* c.1416T>A p.(Y472*)	[56]	Nonsense mutation predicted to result in nonsense-mediated decay	ABCB11 c.1416T>A p.(Y472*)	[56]	Nonsense mutation predicted to result in nonsense-mediated decay	PFIC type 2	C; H; ALF	Not available
97	*ABCB11* c.1445A>G p.(D482G)	[23] (rs72549402)	AGVGD C65 SIFT Deleterious PP Probably damaging SSF No changes MES Cryptic donor NNS No changes GS No changes HSF Cryptic donor	*ABCB11* c.1445A>G p.(D482G)	[23] (rs72549402)	AGVGD C65 SIFT Deleterious PP Probably damaging SSF No changes MES Cryptic donor NNS No changes GS No changes HSF Cryptic donor	PFIC type 2	C; S; H	PEBD at 9 m C resolved
65	*ABCB11* c.1676T>C p.(M559T)	Novel	AGVGD C0 SIFT Deleterious PP Probably damaging SSF No changes MES No changes NNS No changes GS No changes HSF Cryptic donor	*ABCB11* c.3933C>G p.(Y1311*)	Novel	Nonsense mutation predicted to result in nonsense-mediated decay	PFIC type 2	C; S; H; ALF	LT Asymptomatic post-transplant
209	*ABCB11* c.2708T>G p.(V903G)	Novel	AGVGD C35 SIFT Deleterious PP Benign SSF No changes MES No changes NNS No changes GS No changes HSF No changes	*ABCB11* c.2708T>G p.(V903G)	Novel	AGVGD C35 SIFT Deleterious PP Benign SSF No changes MES No changes NNS No changes GS No changes HSF No changes	PFIC type 2	C; S; H; ALF	Not available
26	*ABCB11* c.3517A>G p.(N1173D)	[57]	AGVGD C0 SIFT Deleterious PP Probably damaging SSF No changes MES No changes NNS No changes GS No changes HSF No changes	*ABCB11* c.3628A>C p.(T1210F)	[55,58,59]	AGVGD C0 SIFT Deleterious PP Probably damaging SSF No changes MES No changes NNS No changes GS No changes HSF No changes	PFIC type 2	C	LT Asymptomatic post-transplant
195	*ABCB11* c.3904G>T p.(E1302*)	[55]	Nonsense mutation predicted to result in nonsense-mediated decay	*ABCB11* c.3904G>T p.(E1302*)	[55]	Nonsense mutation predicted to result in nonsense-mediated decay	PFIC type 2	C; S; H	Not available
168	*ABCB4* c.1230+1G>T	Novel	SSF donor destroyed MES donor destroyed NNS donor destroyed GS donor destroyed HSF donor destroyed	*ABCB4* c.1230+1G>T	Novel	SSF donor destroyed MES donor destroyed NNS donor destroyed GS donor destroyed HSF donor destroyed	PFIC type 3	C; S; H; ALF	Not available
204	*ABCB4* c.1624G>C p.(A542P)	Novel	AGVGD C25 SIFT Deleterious PP Possibly damaging SSF No changes MES No changes NNS No changes GS No changes HSF No changes	*ABCB4* c.1624G>C p.(A542P)	Novel	AGVGD C25 SIFT Deleterious PP Possibly damaging SSF No changes MES No changes NNS No changes GS No changes HSF No changes	PFIC type 3	C; H	Not available
36	*ABCB4* c.1652C>T p.(P551L)	Novel	AGVGD C65 SIFT Deleterious PP Probably damaging SSF No changes MES No changes NNS No changes GS No changes HSF No changes	*ABCB4* c.1652C>T p.(P551L)	Novel	AGVGD C65 SIFT Deleterious PP Probably damaging SSF No changes MES No changes NNS No changes GS No changes HSF No changes	PFIC type 3	C; H; ALF	LT Asymptomatic post-transplant
162	*ABCB4* c.1858_1860delAAG p.(K620del)	Novel	SSF No changes MES No changes NNS No changes GS No changes HSF No changes	*ABCB4* c.1858_1860delAAG p.(K620del)	Novel	SSF No changes MES No changes NNS No changes GS No changes HSF No changes	PFIC type 3	H	Not available

Variant interpretation was performed using Alamut v2.1 (Interactive Biosoftware, Rouen, France), which allowed predictions of the effect on protein structure and mRNA splicing using several tools. Protein prediction tools included: Align GVGD (AGVGD); Sorting Intolerant from Tolerant (SIFT); and PolyPhen-2 (PP). Results from AGVGD comprised a spectrum from C0 to C65, with C0 least likely to interfere with function and C65 most likely to interfere with function. Splicing prediction tools included: SpliceSiteFinder-like (SSF); MaxEntScan (MES); NNSplice (NNS); Genesplicer (GS); and Human Splicing Finder (HSF). Results from splicing prediction included: ‘no changes’ (i.e., no change compared with wild-type sequence); ‘donor/acceptor destroyed’ (i.e., predicted loss of wild-type splice site); ‘cryptic donor/acceptor’ (i.e., predicting creation of a novel splice site). Other abbreviations: ALF, acute liver failure; C, cholestasis; H, hepatomegaly; LT, liver transplant; PEBD, partial extrahepatic biliary diversion; S, splenomegaly.

**Table 3:** Genetic findings, presenting features and outcomes in patients with only single heterozygous mutations.

Gene	Mutation	Protein Prediction Tools	Splicing Prediction Tools	Novel or Reference	Patient	Presenting Features	Final Diagnosis and Status at Follow Up
*NPC1*	c.467T>C p.(M156T)	AGVGD C25 SIFT Deleterious PP benign	SSF No changes MES No changes NNS No changes GS No changes HSF No changes	(rs147615070)	104	C; H; S	Symptoms resolved No intervention Alternative diagnosis: multisystem juvenile xanthogranuloma
*NPC1*	c.873G>T p.(W291C)	AGVGD C15 SIFT Deleterious PP Probably damaging	SSF No changes MES No changes NNS No changes GS No changes HSF No changes	(rs138151007)	52	C	Symptoms resolved No intervention
*NPC1*	c.2010C>A p.(C670*)	Nonsense mutation predicted to result in nonsense-mediated decay	–	Novel	4	C; H	Progressive liver disease Alternative diagnosis: BA LT (22 m) Asymptomatic post-transplant
*NPC1*	c.2010C>A p.(C670*)	Nonsense mutation predicted to result in nonsense-mediated decay	–	Novel	10	C; S	Symptoms resolved No intervention Alternative diagnosis: Hirschsprung’s disease necessitating parenteral nutrition.
*NPC1*	c.3107C>T p.(T1036M)	AGVGD C15 SIFT Deleterious PP Probably damaging	SSF No changes MES No changes NNS No changes GS No changes HSF No changes	[60] (rs28942104)	103	C; H	Symptoms resolved No intervention
*NPC1*	c.3614C>T p.(T1205I)	AGVGD C65 SIFT Deleterious PP Probably damaging	SSF Cryptic acceptor MES No changes NNS No changes GS No changes HSF No changes	Novel	47	C; H	Progressive liver disease with portal hypertension
*NPC1*	c.3614C>T p.(T1205I)	AGVGD C65 SIFT Deleterious PP Probably damaging	SSF Cryptic acceptor MES No changes NNS No changes GS No changes HSF No changes	Novel	71	C; H; ALF	Progressive liver disease LT Asymptomatic post-transplant
*ATP8B1*	c.287C>G p.(A96G)	AGVGD C0 SIFT Deleterious PP Possibly damaging	SSF No changes MES No changes NNS No changes GS No changes HSF No changes	Novel	54	C; H	Symptoms resolved.
*ATP8B1*	c.2425A>C p.(I809L)	AGVGD C0 SIFT Deleterious PP Probably damaging	SSF No changes MES No changes NNS No changes GS No changes HSF No changes	Novel	12	C	Progressive liver disease Alternative diagnosis: BA LT (15 m) Asymptomatic post-transplant
*ATP8B1*	c.3043T>C p.(F1015L)	AGVGD C15 SIFT Deleterious PP Probably damaging	SSF No changes MES No changes NNS No changes GS No changes HSF No changes	Novel	32	C; H; S; ALF	Progressive Liver disease Alternative diagnosis: BA LT (10 m) Asymptomatic post-transplant
*ATP8B1*	c.3633C>A p.(F1211L)	AGVGD C15 SIFT Deleterious PP Probably damaging	SSF No changes MES No changes NNS No changes GS No changes HSF No changes	Novel	5	C	Symptoms resolved No intervention
*ATP8B1*	c.3656A>G p.(D1219G)	AGVGD C65 SIFT Deleterious PP Probably damaging	SSF No changes MES No changes NNS No changes GS No changes HSF No changes	Novel	15	C	Progressive liver disease LT(30 m) Asymptomatic post-transplant
*ABCB11*	c.1445A>G p.(D482G)	AGVGD C65 SIFT Deleterious PP Probably damaging	SSF No changes MES Cryptic donor NNS No changes GS No changes HSF Cryptic donor	[23] (rs72549402)	206	C; ALF	PEBD at 5 m Symptoms resolved
*ABCB11*	c.1558A>T p.(R520*)	Nonsense mutation predicted to result in nonsense-mediated decay	–		100	C; H; S	Symptoms resolved No intervention
*ABCB4*	c.3317A>G p.(E1106G)	AGVGD C0 SIFT Deleterious PP Possibly damaging	SSF Cryptic acceptor MES Cryptic acceptor NNS No changes GS No changes HSF No changes	[23] (rs139042803)			
*ABCB11*	c.1621A>C p.(I541L)	AGVGD C0 SIFT Deleterious PP Probably damaging	SSF No changes MES No changes NNS No changes GS No changes HSF No changes	[58,61]	208	C	Symptoms resolved No intervention
*ABCB11*	c.2678C>T p.(A893V)	AGVGD C0 SIFT Deleterious PP Probably damaging	SSF No changes MES No changes NNS No changes GS No changes HSF No changes	Novel	190	C; H; S	Not available
*ABCB4*	c.524C>T p.(T175M)	AGVGD C65 SIFT Deleterious PP Probably damaging	SSF Cryptic acceptor MES No changes NNS No changes GS No changes HSF No changes	Novel	181	C; H	Not available
*ABCB4*	c.1529A>G p.(N510S)	AGVGD C0 SIFT Deleterious PP Possibly damaging	SSF Cryptic donor MES No changes NNS No changes GS No changes HSF Cryptic acceptor	[62] (rs375315619)	13	C; S	Progressive liver disease Alternative diagnosis: BA LT (8 m) Asymptomatic post-transplant
*ABCB4*	c.3403G>A p.(E1135K)	AGVGD C55 SIFT Deleterious PP Benign	SSF No changes MES No changes NNS No changes GS No changes HSF No changes	Novel	53	C; S; ALF	Progressive liver disease (multi-organ failure) Died age 15 m
*SLC25A13*	c.1903G>T p.(D635Y)	AGVGD C15 SIFT Deleterious PP Possibly damaging	SSF No changes MES No changes NNS No changes GS No changes HSF No changes	Novel	151	C; H; S	Symptoms resolved No intervention

Variant interpretation was performed using Alamut v2.1 (Interactive Biosoftware, Rouen, France), which allowed predictions of the effect on protein structure and mRNA splicing using several tools. Protein prediction tools included: Align GVGD (AGVGD); Sorting Intolerant from Tolerant (SIFT); and PolyPhen-2 (PP). Splicing prediction tools included: SpliceSiteFinder-like (SSF); MaxEntScan (MES); NNSplice (NNS); Genesplicer (GS); and Human Splicing Finder (HSF). Results from splicing prediction included: ‘no changes’ (i.e., no change compared with wild-type sequence); ‘donor/acceptor destroyed’ (i.e., predicted loss of wild-type splice site); ‘cryptic donor/acceptor’ (i.e., predicting creation of a novel splice site). Other abbreviations: ALF, acute liver failure; C, cholestasis; H, hepatomegaly; LT, liver transplant; PEBD, partial extrahepatic biliary diversion; S, splenomegaly.

## Data Availability

Data from the GeneChip study is available on request.

## Abbreviations

A1AT	α1-antitrypsin
*ABCB4*	ATP binding cassette subfamily B member 4
*ABCB11*	ATP binding cassette subfamily B member 11
AGVGD	Align GVGD
ALF	acute liver failure
*ATP8B1*	ATPase phospholipid transporting 8B1
BA	biliary atresia
BRIC	benign recurrent intrahepatic cholestasis
BSEP	bile salt export pump
DIC	drug-induced cholestasis
DNA	deoxyribonucleic acid
GRACILE	Growth Retardation, Aminoaciduria, Cholestasis, Iron overload, Lactic acidosis, and Early death
GS	Genesplicer
HSF	Human Splicing Finder
IBAT	ileal bile acid transporter
ICP	intrahepatic cholestasis of pregnancy
*KIF12*	kinesin family member 12
LT	liver transplant
LPAC	low phospholipid-associated cholelithiasis
*LSR*	lipolysis stimulated lipoprotein receptor
MDR3	multidrug resistance protein 3
MES	MaxEntScan
MS	microarray resequencing
*MYO5B*	myosin VB
NGS	next-generation sequencing
NICCD	neonatal intrahepatic cholestasis due to citrin deficiency
NNS	NNSplice
NPC	Niemann–Pick disease type C
NPC-1	Niemann–Pick disease type C1
*NPC1*	NPC intracellular cholesterol transporter 1
*NPC2*	NPC intracellular cholesterol transporter 2
PEBD	partial extrahepatic biliary diversion
PFIC	progressive familial intrahepatic cholestasis
PP	PolyPhen-2
RAB	Ras-related in brain
RNA	ribonucleic acid
SIFT	Sorting Intolerant from Tolerant
*SLC25A13*	solute carrier family 25 member 13
SSF	SpliceSiteFinder-like
TNC	transient neonatal cholestasis
